# One-step endoscopic ultrasound-guided fine-needle biopsy of pancreatic mass, gastroenterostomy, and gallbladder drainage for malignant biliary and gastric outlet obstruction

**DOI:** 10.1055/a-2127-4436

**Published:** 2023-08-01

**Authors:** Benedetto Mangiavillano, Luca Brandaleone, Francesco Auriemma, Federica Calabrese, Danilo Paduano, Carmine S. Gentile, Alessandro Repici

**Affiliations:** 1 Gastrointestinal Endoscopy Unit, Humanitas Mater Domini, Castellanza, Varese, Italy; 2 Endoscopy Unit, Humanitas Clinical and Research Center – IRCCS, Rozzano, Milan, Italy; 3 Humanitas University, Department of Biomedical Sciences, Pieve Emanuele, Milan, Italy

A 77-year-old woman was admitted to our emergency department because of a 2-week history of vomiting, weight loss, and abdominal pain. An abdominal computed tomography (CT) scan revealed a 4-cm pancreatic mass that resulted in gastric outlet obstruction and dilation of the biliary tree and gallbladder. In a multidisciplinary meeting, it was decided to perform endoscopic ultrasound (EUS)-guided fine-needle biopsy (EUS-FNB), EUS-guided gastroenterostomy (EUS-GE), and EUS-guided gallbladder drainage (EUS-GBD) using two lumen-apposing metal stents (LAMSs). Under general anesthesia, EUS-FNB of the pancreatic mass was performed through the stomach using a 22-G needle (Acquire; Boston Scientific, Natick, Massachusetts, USA) ( Video 1 ). Subsequently, a nasobiliary tube was passed through the neoplastic stricture to fill the jejunal loop with saline solution, methylene blue, and contrast medium. A 16 × 20-mm electrocautery-enhanced LAMS (Hot-Spaxus; Taewoong Medical, Gimpo-si, Korea) was passed from the stomach to the dilated jejunal loop using the freehand technique ( Fig. 1 ). The proximal flange of the LAMS was deployed using the intrachannel technique. Subsequently, EUS-GBD was performed using an 8 × 20-mm electrocautery-enhanced LAMS (Hot-Spaxus; Taewoong Medical) and the freehand technique ( Fig. 2 ). No adverse events were experienced by the patient. The post-procedural CT scan confirmed the accurate positioning of the two LAMSs between the stomach and jejunum, and between the stomach and gallbladder. The patient was discharged after 3 days, having successfully resumed oral feeding. In conclusion, in such cases, a one-step approach involving EUS-FNB, EUS-GE, and EUS-GBD is safe when performed by experts ( Video 1 ).

**Video 1** One-step approach involving endoscopic ultrasound (EUS)-guided fine-needle biopsy of pancreatic neoplasia, EUS-guided gastroenterostomy (EUS-GE), and EUS-guided cholecystogastrostomy (EUS-CSG). NBD, nasobiliary tube.


**Fig. 1:**
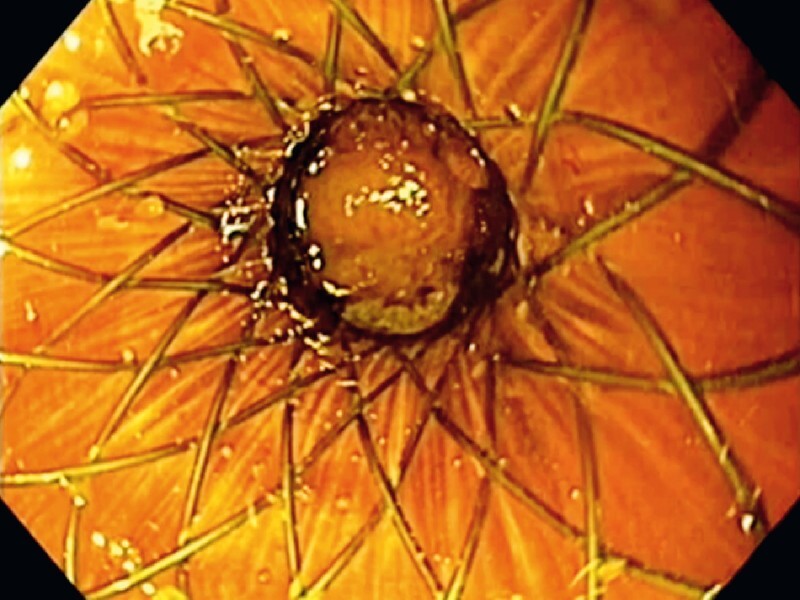
Placement of an 16 × 20-mm electrocautery-enhanced lumen-apposing metal stent during endoscopic ultrasound-guided gastroenterostomy.

**Fig. 2:**
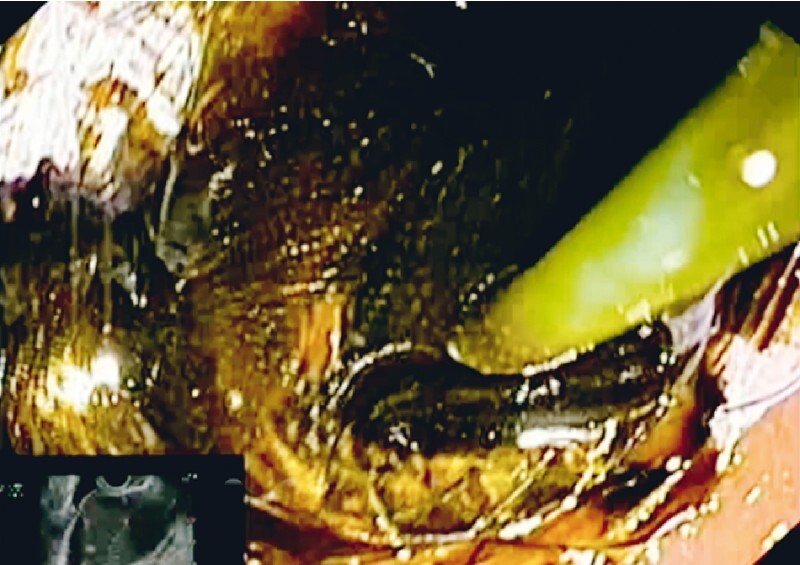
Endoscopic ultrasound-guided cholecystogastrostomy with placement of a 8 × 20-mm electrocautery-enhanced lumen-apposing metal stent, resulting in appropriate biliary drainage.

EUS-GE is a novel endoscopic technique that has comparable technical and clinical success rates to surgical gastroenterostomy. It is useful for patients who are not suitable for surgery because of frailty or when a temporary bridging measure is required before surgery 1 2 . In addition to EUS-GE, EUS-GBD serves as an alternative approach for managing biliary tree and gallbladder dilatation in patients who are deemed unfit or at high risk for surgery. This technique has shown comparable clinical and technical success rates to percutaneous drainage.

Endoscopy_UCTN_Code_CPL_1AL_2AD

## Notes

Endoscopy E-Videos

https://eref.thieme.de/e-videos
*E-Videos* is an open access online section of the journal *Endoscopy* , reporting on interesting cases and new techniques in gastroenterological endoscopy. All papers include a high-quality video and are published with a Creative Commons CC-BY license. Endoscopy E-Videos qualify for HINARI discounts and waivers and eligibility is automatically checked during the submission process. We grant 100% waivers to articles whose corresponding authors are based in Group A countries and 50% waivers to those who are based in Group B countries as classified by Research4Life (see: https://www.research4life.org/access/eligibility/ ). This section has its own submission website at https://mc.manuscriptcentral.com/e-videos
